# Middle Cerebral Artery Pulsatility Index as Predictor of Cognitive Impairment in Hypertensive Patients

**DOI:** 10.3389/fneur.2018.00538

**Published:** 2018-07-20

**Authors:** Salim Harris, Teuku Reyhan, Yetty Ramli, Joedo Prihartono, Mohammad Kurniawan

**Affiliations:** ^1^Department of Neurology, Faculty of Medicine, Universitas Indonesia, Jakarta, Indonesia; ^2^Department of Community Medicine, Faculty of Medicine Universitas Indonesia, Jakarta, Indonesia

**Keywords:** cognitive impairment, hypertension, middle cerebral artery, pulsatility index, trans cranial doppler

## Abstract

**Background:** Cognitive impairment is a manifestation of cerebrovascular disease regarding hypertension and other degenerative diseases which has become a global health issue due to increased life expectancy. Recently, the gold standard used for diagnosing vascular cognitive impairment (VCI) has required a combination of the neurophysiological approach and magnetic resonance imaging (MRI). The Neurosonological approach, involving measuring the pulsatility index (PI) of the middle cerebral artery (MCA) using Trans Cranial Doppler (TCD), can hopefully be used as an affordable alternative predictor of VCI in patients with hypertension.

**Methods:** A cross-sectional study was conducted at the Outpatient Clinic of the Department of Neurology in Ciptomangunkusumo Hospital, Jakarta. Sixty-six hypertensive subjects with no macrovascular complication were selected and screened using the Montreal Cognitive Assesment-Indonesia version (MoCA-Ina) to determine their cognitive status. Subjects were categorized into two groups; subjects with scores ≥26 were classified as the normal cognitive group, while subjects with scores <26 were classified as the cognitive impairment group. Both groups then underwent TCD examination to determine bilateral MCA PI.

**Results:** There was a significant difference between MCA PI in both groups; it was higher in the cognitive impairment group than normal group (*p* < 0.001). Subjects with an increased left MCA PI were more likely to suffer cognitive impairment than those with an increased right MCA PI.

**Conclusion:** MCA PI can be used as a predictor for cognitive impairment in hypertensive subjects.

## Introduction

Cognitive function is a complex system. It consists of several domains such as attention, language, memory, visuospatial, and executive functions (1). An impaired cognitive function was considered as a serious global issue, especially today where life expectancy is high (2). One of the most important risk factors of cognitive impairment is cerebrovascular disease, particularly hypertension (3). Cognitive impairment caused by vascular disease is known as the vascular cognitive impairment (VCI). VCI can lead to vascular dementia (VaD), which is one of the main causes of dementia in Asia (4). It is estimated that 1.5–4.8% people in their 70s suffered from dementia globally, and this number is growing with age. In the US, it is estimated that 9–36% of people aged over 65 suffer from dementia (5). Meanwhile, hypertension as the leading cause of VCI also has a high prevalence worldwide. In Indonesia, the prevalence of hypertension in adults aged over 18 was 25.8% in 2013 (6).

Hypertension along with type-2 diabetes mellitus (T2DM) and dyslipidemia promote the formation of arteriolosclerosis, which increased peripheral vascular resistance and causing hypoperfusion on the affected area. Ultimately, it leads to VCI (6, 7). The most common domains affected by VCI are executive function and memory (5).

The gold standard diagnosis for VCI requires a combination of neuropsychological examinations (such as the Montreal Cognitive Assessment questionnaire, which is more sensitive to evaluate executive function compared to other neuropsychological instruments (8)) and brain Magnetic Resonance Imaging (MRI), to determine the area of infarct (5, 9). The presence of abnormality in MRI, such as hypointensity in T1-weighted image and white matter lesions in T2-FLAIR, can also be used as a predictor of cognitive impairment because of brain hypoperfusion (10, 11); however, MRI has its own challenges as it is less available and relatively expensive. Therefore, an affordable alternative diagnostic method is needed.

The Trans Cranial Doppler (TCD) has been used to evaluate the hemodynamics of basal cranial blood vessels (middle cerebral artery, anterior cerebral artery, posterior cerebral artery, vertebral artery and basilar artery). One of the parameters of TCD is Pulsatility Index (PI), which evaluates the peripheral resistance of blood vessels. Increased PI can indicate an increased resistance in the distal area of the blood vessel, which shows that there is hypoperfusion in the area (12). It has been estimated that increased middle cerebral artery (MCA) PI in hypertensive patients can be a predictor of arteriolosclerosis, which is the main cause of VCI, but has not yet concluded. Therefore, this study aims to evaluate whether MCA PI can be a predictor of VCI compared to the gold standard diagnostic method.

## Method

A cross-sectional study was done to all hypertensive patients who had been seeking treatment for at least 4 years at the outpatient clinics of Department of Neurology at Cipto Mangunkusumo National Central General Hospital between January and April 2016. The inclusion criteria were (1) patients aged between 18 and 65 years; (2) patients with clinical diagnosis of hypertension as stated in the JNC 7/8 classification with minimum duration of 4 years; (3) patients who were willing to participate in the study. The exclusion criteria were those who were experiencing or had a history of stroke, epilepsy, Parkinson disease, brain tumor, brain infection, chronic kidney failure, congestive heart failure, malignancy or mental disorder.

The Pulsatility index (PI) was measured to predict stenosis on the blood vessel proximal to the measurement site (13). PI was measured by certified neurologists in Cipto Mangunkusumo Hospital using Rimed Digi-Lite™; which was calculated automatically using this formula:

$$\begin{array}{l} PI=\frac{PSV-EDV}{MV} \end{array}$$

PSV = peak systolic velocity, EDV = end-diastolic volume, and MV = mean flow velocity during cardiac cycle (14, 15).

Cognitive impairment was assessed using the Indonesian version of neuropsychological examination (MOCA-Ina) by certified neurologists. Subjects with a MOCA-Ina score <26 were classified as cognitive impairment while subjects with a score ≥26 were classified as normal. All subjects in both groups underwent TCD examination in which bilateral MCA PI was evaluated. MCA was used because it could represent a total blood flow in the hemisphere, considering high blood volume (60–70%) flowed to MCA from ICA (16). In fact, recent study showed that vasospasm evaluation of the MCA was more reliable (99% specific and 67% sensitive) compared to PCA (48% sensitive and 69% specific) and ACA (18% sensitive and 65% specific) (14).

Statistical analysis was conducted using SPSS 17. Normality data was tested using Saphiro-Wilk. Descriptive analysis was done using Chi-Square and Unpaired *T*-test. If data was not distributed normally, the analysis was continued using Mann-Whitney or Wilcoxon. The correlative test was done using Pearson for normally distributed data, and Spearman if data were not distributed normally.

## Results

We enrolled 66 subjects that matched our criteria. Subjects were classified into two groups; 32 without cognitive impairment (48.5%) and 34 with cognitive impairment (51.5%). Demographic characteristics of all subjects were presented in Tables 1, 2, there was no significant difference between both groups (*p* > 0.05). However, there were significant differences of mean MCA PI between normal and impaired cognitive function groups in both arteries as presented in Tables 3, 4 (*p* < 0.05).

**Table 1:** Demographic characteristics.

**Demographic characteristics**	**Cognitive**		*p*-value^*^
	**Normal**	**Impaired**	
**Sex**			
Male	16	14	0.637
Female	16	20	
**Diabetes**			
Yes	8	17	0.066
No	24	17	
**Smoking**			
Yes	11	13	0.944
No	21	21	
**Treatment using ace inhibitor**			
Yes	12	17	0.439
No	20	17	
**Treatment using angiotensin receptor blocker**			
Yes	6	5	0.912
No	26	29	
**Treatment using calcium channel blocker**			
Yes	12	20	0.137
No	20	14	

* *Chi-Square test*.

**Table 2:** Age characteristics and duration of hypertension.

**Demographic characteristics**	**Normal Cognitive**		**Impaired Cognitive**		*p*-value^**^
	**Median**	**Range**	**Median**	**Range**	
Subjects' age	51.8	28–65	57.0	38–65	0.059
Duration of hypertension	8.0	4–15	9.5	4–25	0.175

** *Mann Whitney test*.

**Table 3:** Mean value of MCA PI in both groups.

**MCA PI**	**Normal Cognitive**		**Impaired Cognitive**		*p*-value^***^
	**Mean**	***SD***	**Mean**	***SD***	
Right side PI	0.87	0.15	1.18	0.22	< 0.01
Left side PI	0.86	0.18	1.21	0.17	< 0.01

*** *Student T-test*.

**Table 4:** Correlation between MCA PI and cognitive status.

**PI**	**Cognitive**		**P^β^**	**OR**	**95% Confidence Interval**	
	**Normal**	**Impaired**			**Lowest**	**Highest**
**Right MCA PI**
• Normal	30	13	< 0.0001	24.231	4.943	118.78
• Abnormal	2	21				
**Left MCA PI**
• Normal	30	8	< 0.0001	48.75	9.494	250.328
• Abnormal	2	26				
Total	32 subjects	34 subjects				

β *Chi square test*.

In the normal cognitive group, we found that the mean value of right MCA PI was 0.87 (±0.15) and the left MCA PI was 0.86 (±0.18); while in the group with impaired cognitive, we found that the mean value of right MCA PI was 1.18 (±0.22) and left MCA PI was 1.21 (±0.17). Statistical tests were performed to evaluate any significant findings in both groups and we found *p* < 0.0001 for right MCA PI and *p* ≤ 0.0001 for left MCA PI between both groups. Further analysis showed that there was a significant correlation between left and right MCA PI and cognitive status (*p* < 0.0001). Subjects with abnormal right MCA PI were 24.2 times more likely to have cognitive impairment compared to subjects with a normal PI. While subjects with abnormal left MCA PI were 48.7 times more likely to have cognitive impairment compared to subjects with normal PI. The study also showed a 95% confidence interval (CI) of right MCA PI was 4.9–118.8; while on the left side, it was 9.5–250.3.

**Figure 1:**
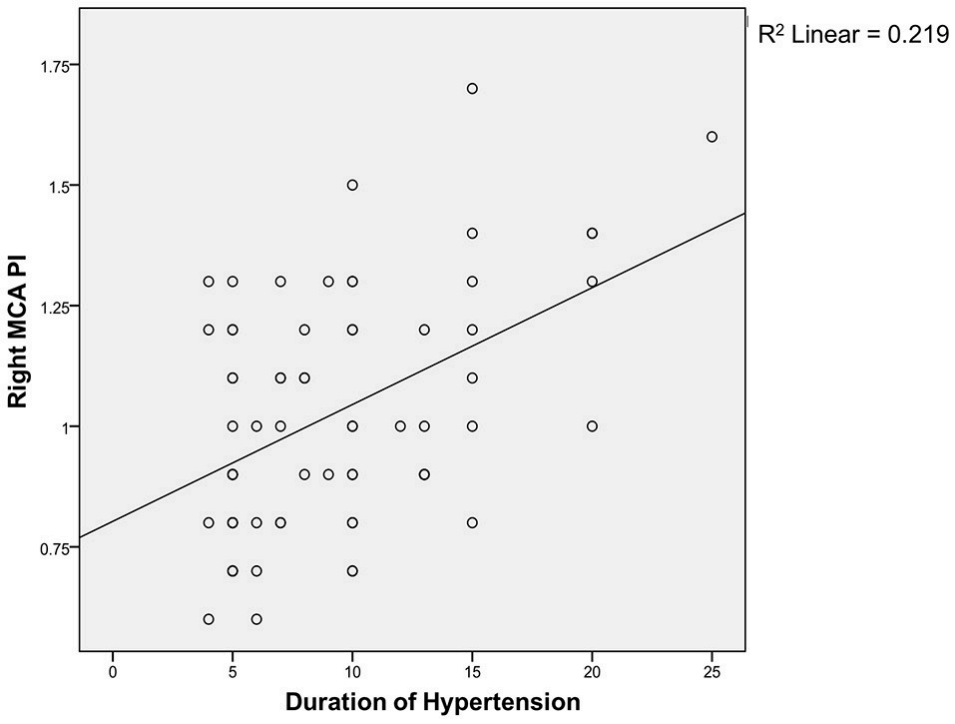
Correlation between duration of hypertension and right MCA PI.

**Figure 2:**
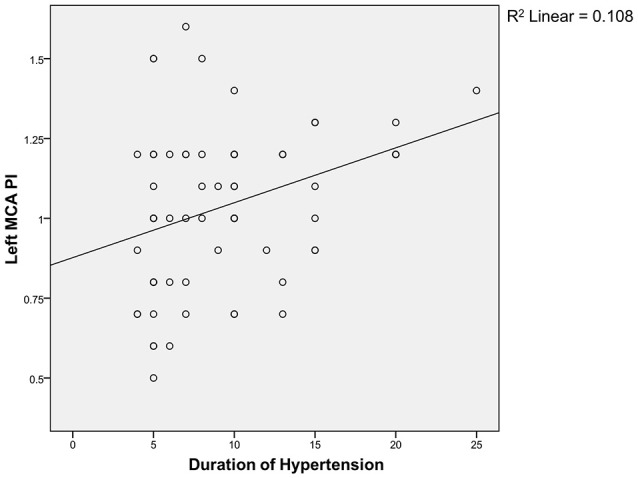
Correlation between duration of hypertension and left MCA PI.

Spearman correlation test (Figures 1, 2) showed that the coefficient of left and right MCA PI was 0.11 and 0.22 respectively. It indicates that there was a weak correlation between the duration of hypertension and increased MCA PI.

## Discussion

In our study, we found that there were 32 subjects (48.5%) without cognitive impairment and 34 subjects (51.5%) with cognitive impairment. There were more female subjects (*n* = 36) than male (*n* = 30). The age range of our subjects was 28 to 65 years old with the mean age of 54.1 ± 8.2. Our results were similar to other studies conducted by Indrasari (55.05 ± 8.38 years), Kurniawan (56.3 ± 7.0 years) and Kalbi (55.4 ± 8.1 years) (17–19).

The Mean duration of hypertension in our study was 9.4 ± 4.8 years. Our result was not different from the similar study conducted by Elias et al, which showed 12.8 ± 5.5 years (9). In our study, we found type 2 Diabetes Mellitus (DM) in 25 subjects, in which 50% of the subjects had cognitive impairment. Hypertension and type 2 DM is a common coexistent condition due to a metabolic disorder which may result in an interconnected cycle.

Hyperinsulinemia in insulin-resistant DM increases the activity of sodium-potassium ATPase canal: thus increasing the intracellular sodium level. Therefore, it will increase contraction of vascular muscles until vasoconstriction occurs (20, 21). DM can occur secondary due to hypertension as a result of defects in cation transport on the membrane; hence, increased sodium level in hypertension will disturb sodium efflux and potassium influx. These ion pump failures cause insulin to be less effective on the cell membrane. In our study, we found no significant difference regarding DM prevalence between both groups.

The correlation between cognitive function and smoking has been widely published. A study in China conducted by Huadong et al. found a significant correlation between smoking and cognitive function (22). Another study by Richards et al. (23) also found a correlation between smoking of more than 20 cigarettes per day and cognitive impairment (23). In our study, we found 24 subjects who were smokers. No significant difference was found in the distribution of smoker subjects between both groups.

The use of anti-hypertensive agents in our study has also been included as a variable of study. Some cohort studies in the post-war era of the 1960's have associated the use of captopril with the risk of cognitive impairment. A study in England in 1967 correlated reduced daily living activity with the use of antihypertensive agents (24). The developing hypothesis suggests that reduced blood pressure causes reduced cerebral blood flow in the area, which is vital for cognitive function. However, some studies in the 1990's have shown contrasting results, which demonstrate the positive role of anti-hypertensive agents in improving cognitive function. Forettie et al. in the Syst-Eur study (2002) conducted a double-blind clinical trial and found that nifedipine, enalapril and hydrochlorothiazide reduce the risk of vascular dementia significantly compared to placebo group (25). In our study, we found no significant difference regarding the utilization of anti-hypertensive agents in both groups.

Our study used TCD instruments to evaluate the condition of right and left basal cranial blood vessels. The measurement was conducted in three transcranial windows (orbital, temporal, suboccipital) to measure vascular parameters at anterior, medial, posterior cerebral arteries and vertebral, as well as basillar arteries. In this study, only PI obtained from the MCA was included in the analysis, considering large portion (two-third) of brain is supplied by the MCA; including the areas responsible for cognitive function. Moreover, the MCA is easily accessed through the temporal window and the long MCA track is suitable for evaluating peripheral resistance in the distal area in which direct examination is hardly performed (12). We did not perform an analysis of the posterior circulation due to lower sensitivity and specificity of posterior circulation compared to MCA, albeit the fact that damage in those areas contribute to the development of cognitive impairment (14, 26, 27); therefore, we acknowledge this as a limitation.

Data analysis on all subjects demonstrated that mean the PI of right MCA was 1.03 ± 0.248 and left MCA was 1.04 ± 0.250. In the group with cognitive function, we found that the mean PI of the right MCA was 1.18 ± 0.22 and the left MCA was 1.21 ± 0.17. Similar results were also found by Vicenzini et al. i.e., in the group with vascular dementia, the PI value of right MCA was 1.19 ± 0.06 and the left MCA was 1.1 ± 0.05 (28). Indrasari has reported that the PI of right MCA in patients with hypertension and DM is 1.17 ± 0.25 (19).

In our study, we found a significant difference of MCA PI between the normal group and the group with cognitive impairment. The significant difference of MCA PI suggests that cognitive impairment may have been caused by increased peripheral resistance, which causes disturbed blood flow that results in damage to the distal area supplied by the MCA. The process is part of small vessel disease which becomes the principle of vascular cognitive impairment (VCI) theory. Lee et al. correlates increased PI in MCA that can describe the microangiopathy process in all of the cerebral arteries (29). The Microangiopathy process also releases some inflammatory cytokines, such as endothelin and prostacyclin, as well as interrupting the autoregulation of cerebral blood flow, which impairs the blood flow to the distal area. Vicenzini et al. have found that increased PI is associated with reduced end-diastolic volume (EDV), which indicates the development of vasoconstriction at the distal of blood vessels (30).

Subjects with an abnormal right MCA PI are 24.2 times more likely to have cognitive impairment compared to subjects with an normal right MCA PI (95%CI = 4.9–118.8). While subjects with an abnormal left MCA PI are 48.7 times more likely to have a cognitive impairment (95%CI = 9.5–250.3).

The different ratio between right and left MCA PI can be explained by the hemisphere theory. The left brain hemisphere has a role in auditory memory, particularly for verbal material, while the right hemisphere is more likely to have a role in non-verbal memory (5, 30). In executive function, difficulties in writing, reading and understanding numbers are commonly associated with aphasia. Aphasia is an abnormality on the non-dominant (left) hemisphere and largely associated with damage in the area of MCA distribution. It explains why the subjects with an abnormal PI of the left side of the MCA have larger possibilities of having cognitive impairment compared to the abnormal PI of the right MCA.

Regarding the correlation between the duration of hypertension and PI, we found a weak correlation between both groups. The weak correlation, in the authors' opinion, is associated with the limitation of our study, i.e., the limited sample size; therefore, it is difficult to evaluate the correlation directly. The duration of hypertension was mostly made based on the patients' estimation and therefore, it is difficult to find an accurate correlation. There is no similar study conducted, both nationwide and worldwide, and so we do not have any comparable data.

## Conclusion

MCA PI can be used as a predictor for the development of cognitive impairment. Increased PI in subjects with normal cognitive functions can be a justification for aggressive therapy and periodic evaluation on cognitive function, while PI in subjects with cognitive impairment can be an indicator to measure therapeutical effectiveness and may act as a predictor for impaired cognitive function. There is a weak correlation between the duration of hypertension and PI; therefore, further studies should be conducted to discover other factors which may affect PI.

## Ethics statement

The protocol was approved by the Health Research Ethics Committee, Faculty of Medicine, Universitas Indonesia-Cipto Mangunkusumo Hospital. All subjects gave written informed consent in accordance with the Declaration of Helsinki.

## Author contributions

SH and TR contributed to the conception of the design of the study. SH, TR, YR, and MK collected all the data and supporting articles. SH, JP, and TR analyzed the data. All authors helped with writing the manuscript.

### Conflict of interest statement

The authors declare that the research was conducted in the absence of any commercial or financial relationships that could be construed as a potential conflict of interest.
